# MYD88‐mutated Lymphoplasmacytic Lymphoma With Monoclonal Immunoglobulin G: A Case Report

**DOI:** 10.1002/jha2.70027

**Published:** 2025-03-27

**Authors:** Morten Yung Isaksen, Olav Karsten Vintermyr, Håkon Reikvam

**Affiliations:** ^1^ Department of Medicine Haukeland University Hospital Bergen Norway; ^2^ K.G. Jebsen Center for Myeloid Malignancies Department of Clinical Science University of Bergen Bergen Norway; ^3^ Department of Pathology Haukeland University Hospital Bergen Norway; ^4^ The Gade Laboratory for Pathology Department of Clinical Medicine University of Bergen Bergen Norway

**Keywords:** anaemia | case report | lymphoplasmacytic lymphoma | monoclonal IgG | MYD88 mutation

## Abstract

Lymphoplasmacytic lymphomas (LPL) are usually associated with serum monoclonal immunoglobulin M (IgM). Nevertheless, in some cases, these cells may secrete IgA or IgG monoclonal proteins or remain non‐secretory. We report a case from a patient with LPL‐secreting IgG who developed anaemia and splenomegaly during the disease course that necessitated treatment with bortezomib, dexamethasone, and rituximab. The case illustrates the need for clinicians and pathologists to consider LPLs as a differential diagnosis also without a serum monoclonal IgM.

**Clinical Trial Registration**: The authors have confirmed clinical trial registration is not needed for this submission.

## 1 Introduction

Lymphoplasmacytic lymphomas (LPLs) are indolent B‐cell lymphomas. Most LPLs are associated with clonal lymphoplasmacytic cells secreting immunoglobulin M (IgM), referred to as IgM‐LPL or Waldenström macroglobulinaemia (WM) type. Only about 5% of LPLs are non‐WM type, and cases with monoclonal IgG make up a subset of these. Other non‐WM type LPLs are IgA‐LPLs, non‐secretory LPLs and IgM LPLs with only extramedullary involvement [1].

Compared to WM, non‐WM type LPLs have been shown to present with extramedullary disease more often than WM, while they harbour the *MYD88*‐mutation less frequently than their IgM‐secreting counterpart [2, 3, 4]. Asymptomatic cases are followed with regular observation, while patients with findings suggestive of significant infiltration of the bone marrow or other organs, like cytopenias, splenomegaly, or bulky lymphadenopathy, should be offered therapy. Non‐WM type LPLs are generally treated similarly to classical WM types, with chemoimmunotherapy or a Bruton tyrosine kinase inhibitor [3, 4, 5].

### 1.1 Case Presentation

**FIGURE 1:**
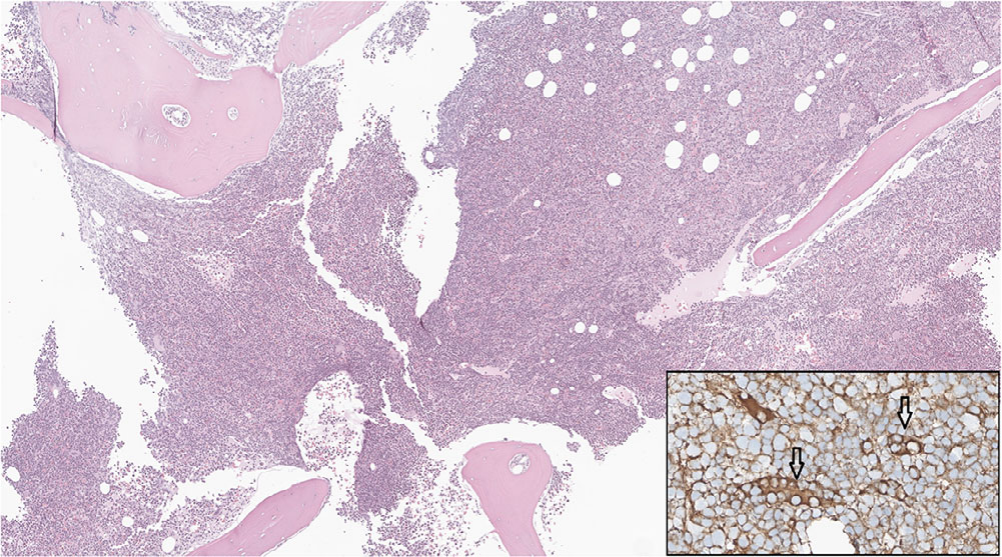
Trephine biopsy showing a hypercellular bone marrow (HE x3). Inset: Plasma cells staining positive for IgG (arrows; IHC x40). HE, haematoxylin‐eosin; IHC, immunohistochemistry.

**FIGURE 2:**
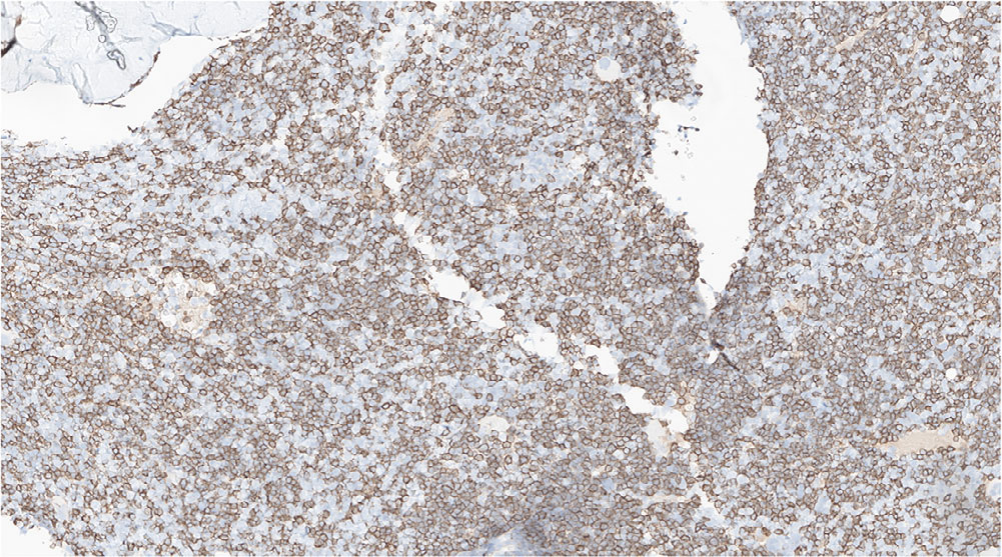
Trephine biopsy with a marked increase of CD20‐positive cells consistent with B‐cells (IHC x8). IHC, immunohistochemistry.

A woman in her 60s was referred to the haematology department due to an elevated erythrocyte sedimentation rate and the presence of a serum IgG monoclonal protein, type kappa. Blood values upon referral are given in Table 1. Bone marrow smear microscopy, performed under suspicion of multiple myeloma, revealed no plasma cells, although it showed a marked increase in lymphocytes, constituting 67% of nucleated cells. Given the findings, lymphoma was suspected. Multiparametric flow cytometry of bone marrow aspirate confirmed a clonal expansion of a distinct cell population with the following immune phenotype: CD45+/19+/20++/23‐/22dim/5‐/10+/38‐/11c‐/kappa light chain positive, comprising approximately 33% of nucleated cells. A subsequent trephine biopsy demonstrated widespread marrow infiltration by lymphoplasmacytic cells in a diffuse and interstitial pattern (Figure 1‐2). Based on these findings, a diagnosis of lymphoplasmacytic lymphoma with monoclonal IgG was established. Further genetic testing revealed the presence of the MYD88 L265P mutation, supporting the diagnosis.

**TABLE 1:** The table demonstrates the most important blood values at the time of diagnosis, initiating of treatment and end of treatment.

Value	Reference	Diagnosis	Initiating treatment	Ending treatment
Haemoglobin	11.7–15.3 g/dL	12.8	9.0	11.4
WBC	4.1–9.8 × 10^9^/L	7.4	19.8	5.6
Platelets	165–387 × 10^9^/L	202	79	172
Creatinine	45–90 µg/L	65	97	86
LDH	115–255 U/L	191	189	183
IgG	6.00–15.3 g/L	45.0	39.7	28.2
IgA	0.80–4.00 g/L	0.5	0.35	0.84
IgM	0.30–2.30 g/L	0.2	0.57	1.27
M‐protein	0.0 g/L	—	21.7	7.5
Kappa light chains	8.30–27.0 mg/L	—	649	382
Lambda light chains	0.31–1.56 mg/L	—	72.9	67.5

Abbreviations: IgA, immunoglobulin A; LDH, lactate dehydrogenase; WBC, white blood cells.

The patient did not have any cytopenias, lymphadenopathy or constitutional symptoms at the time of diagnosis. She was followed untreated with regular visits at the outpatient clinic over several years. More than 5 years after initial diagnosis her haemoglobin levels started to drop, with a slowly worsening anaemia developing over a period of another 7 years. The blood samples at this time are given in Table 1. In addition, the patient had a computed tomography scan of the neck, thoracic‐, abdominal‐, and pelvic cavity, which showed an enlarged spleen with a diameter of 21 cm at the most and a couple of slightly enlarged lymph nodes in the mediastinum. A repeated trephine biopsy was done with similar findings as at the time of initial diagnosis.

The patient had symptoms of anaemia and discomfort from her enlarged spleen, and hence indication for starting therapy for her LPL was found. Treatment with bortezomib, dexamethasone, and rituximab (BDR) as first‐line treatment was initiated. After six cycles of BDR the patient was in good clinical condition. Her peripheral haematological values had almost normalized (Table 1). M protein levels were reduced to 7.5 g/L consistent with a partial response to therapy. The patient is currently followed untreated with regular visits at the outpatient clinic. The M protein levels are gradually rising, although she has so far not developed any signs indicating the need for second‐line therapy.

## 2 Discussion

LPLs are rare lymphomas, making up approximately 2% of haematological malignancies in Europe and the United States annually [6, 7]. As a rare subgroup, LPLs associated with monoclonal proteins other than IgM make up only a small fraction of the total cases of LPLs. Previous case series and register‐based studies have shown clinical differences between non‐WM LPL and WM, but the total number of patients with non‐WM LPL is small, and there is considerable heterogeneity between findings in the reports. When comparing cases of non‐WM LPL to WM, several studies found significantly more extramedullary symptoms in patients with non‐WM LPL, while the *MYD88*‐mutation was less frequently found in these patients. On the other hand, there were inconsistent findings regarding differences in age, sex and laboratory findings at the time of diagnosis across the non‐WM LPL and WM groups [2–4, 9, 10]. Due to the natural absence of elevated levels of the high molecular weight IgM‐pentamer, patients with non‐WM LPL rarely show symptoms of hyperviscosity.

In our patient, the findings of widespread marrow infiltration by lymphoplasmacytic cells in the trephine biopsy suggested an LPL. Alternative diagnoses were considered, like marginal zone lymphoma and follicular lymphoma, but immunohistochemical findings were not consistent with either of these. The finding of a *MYD88 L*265P mutation in our patient also supported the diagnosis of an LPL. The patient was not tested for *CXCR4*‐mutations, the second most common somatic mutation in patients with WM.

Our patient developed symptomatic splenomegaly and anaemia more than 10 years after the initial diagnosis. Due to its rarity, there are no guidelines specific to the treatment of non‐WM LPLs, and they are often treated similarly to WM. Several reports comparing non‐WM type LPLs to WM have found a shorter time from diagnosis to first treatment for patients with non‐WM type LPLs [2, 3].

For our patient, a regimen consisting of BDR was chosen above bendamustine and rituximab, as the indication for therapy was bone marrow failure, and the BDR regime is regarded as less myelosuppressive [8]. She showed good tolerance for treatment with BDR and is currently feeling well more than 18 months after finishing therapy.

Overall survival (OS) in patients with non‐WM type LPLs seems to be similar to OS in patients with WM [2, 3, 9]. Even though non‐WM LPLs share several features with classical WM, this subtype of LPL is not well understood. Further research is warranted to discriminate unique clinical, pathological and molecular features of non‐WM type LPL, as well as disease‐specific therapy and follow‐up.

## Author Contributions

All authors have contributed to the manuscript and approved the final version.

## Conflicts of Interest

The authors have nothing to report.

## Patient Consent Statement

Supplied upon request
